# Evaluation of psychological stress, cortisol awakening response, and heart rate variability in patients with chronic prostatitis/chronic pelvic pain syndrome complicated by lower urinary tract symptoms and erectile dysfunction

**DOI:** 10.3389/fpsyg.2022.903250

**Published:** 2022-11-04

**Authors:** Jian Bai, Longjie Gu, Yinwei Chen, Xiaming Liu, Jun Yang, Mingchao Li, Xiyuan Dong, Shulin Yang, Bo Huang, Tao Wang, Lei Jin, Jihong Liu, Shaogang Wang

**Affiliations:** ^1^Reproductive Medicine Center, Tongji Hospital, Tongji Medical College, Huazhong University of Science and Technology, Wuhan, China; ^2^Department of Urology, Tongji Hospital, Tongji Medical College, Huazhong University of Science and Technology, Wuhan, China

**Keywords:** prostatitis, lower urinary tract symptoms, erectile dysfunction, stress, cortisol, heart rate variability

## Abstract

**Background:**

Mental stress and imbalance of its two neural stress systems, the autonomic nervous system (ANS) and the hypothalamic–pituitary–adrenal (HPA) axis, are associated with chronic prostatitis/chronic pelvic pain syndrome (CP/CPPS) and erectile dysfunction (ED). However, the comprehensive analyses of psychological stress and stress systems are under-investigated, particularly in CP/CPPS patients complicated by lower urinary tract symptoms (LUTS) and ED.

**Materials and methods:**

Participants were 95 patients in CP/CPPS+ED group, 290 patients in CP/CPPS group, 124 patients in ED group and 52 healthy men in control group. The National Institutes of Health Chronic Prostatitis Symptom Index (NIH-CPSI), the International Index of Erectile Function-5 (IIEF-5) and the International Prostate Symptom Score (IPSS) were used for assessing the disease severity of CP/CPPS, LUTS and ED. Psychometric self-report questionnaires including the Beck Anxiety Inventory (BAI), Perceived Stress Scale (PSS), Type A Personality Test (TAPT) and Symptom Checklist 90 (SCL-90) were completed for distress from physical symptoms. Twenty-five subjects per group were randomly selected for further investigating the changes of the HPA axis and ANS. Saliva samples were taken on 3 consecutive days at 8 specific times with strict reference to time of morning awakening for evaluation of free cortisol. Heart rate variability (HRV) as marker of the ANS was measured using 24 h electrocardiography, and time-and frequency-domain variables were analyzed.

**Results:**

The BAI and SCL-90 scores were significantly higher in the CP/CPPS+ED, CP/CPPS and ED groups compared with the control group (*p* < 0.01). The PSS scores of both groups with ED were significantly higher than the control group (*p* < 0.01). Compared with the CP/CPPS group, the differences of PSS, SCL-90 and TAPT scores were statistically significant in CP/CPPS+ED patients (*p* < 0.01). The IPSS scores were shown to have significantly positive correlations with BAI (*r* = 0.32, *p* < 0.0001), PSS (*r* = 0.18, *p* < 0.01) and SCL-90 (*r* = 0.19, *p* < 0.01) in the CP/CPPS patients. However, in all subjects, the IIEF-5 scores were shown to have significantly negative correlations with BAI (*r* = −0.17，*p* < 0.001), PSS (*r* = −0.25，*p* < 0.0001), SCL-90 (*r* = −0.20，*p* < 0.001) and quality of life score in NIH-CPSI (*r* = −0.14，*p* = 0.0075). Cortisol awakening response (CAR) parameters and diurnal cortisol levels did not significantly vary between the four groups. Time-dependent parameters of HRV also did not differ significantly across groups. In the frequency domain analysis, low frequency (LF) was significantly lower in ED patients when compared with CP/CPPS+ED patients (*p* = 0.044) and healthy controls (*p* = 0.005), high frequency (HF) power was significantly higher in healthy controls compared to patients with ED (*p* < 0.001), CP/CPPS (*p* < 0.001) and CP/CPPS+ED (*p* < 0.001), and the CP/CPPS+ED group had significantly higher LF/HF ratio than the control group (*p* = 0.001).

**Conclusion:**

CP/CPPS and ED patients score exceedingly high on most psychosocial variables. The symptom scores of LUTS and ED positively correlate with the severity of psychological stress. Our findings also suggest that the ANS sympathovagal imbalance is associated with ED and LUTS in CP/CPPS, whereas HPA axis activity is not.

## Introduction

Chronic prostatitis is a disease that seriously affects the quality of life (QoL) of most patients because it is not only a physical disease but also a psychological distress (Busetto et al., 2014). Chronic prostatitis/chronic pelvic pain syndrome (CP/CPPS) is a condition featuring multiple symptoms such as persistent and widespread pelvic pain, as well as lower urinary tract symptoms (LUTS) without demonstrable voiding tract infection, which can be accompanied by psychosocial or psychiatric comorbidities, severely decreasing the QoL in men (Krieger et al., 1999; Huang et al., 2020). As the most common type of prostatitis (Fan et al., 2021), CP/CPPS prevalence is estimated at 2.2–13.8% in the male population (Zhang et al., 2016). Erectile dysfunction (ED), defined as the inability to attain and maintain satisfactory erections for adequate sexual performance, is closely correlated to CP/CPPS (Alkan et al., 2018). CP/CPPS patients are more likely to experience ED compared with normal population (Hu et al., 2016). The incidence of ED ranges from 13.8 to 48.3% in men with CP/CPPS, and its prevalence continues to increase (Wang et al., 2016). On the other hand, CP/CPPS in ED patients also has a higher incidence rate (Zhang et al., 2016). It has been reported that CP/CPPS was present in 8.6% of patients with ED, significantly higher than in 2.5% of patients without ED, and ED patients are 3.62 times more likely to develop CP/CPPS than those without ED (Chung et al., 2012).

Prostate health is strictly associated with overall male fertility, homeostasis, and general wellness. For example, prostate cancer and its subsequent treatments have a great impact on the patients’ functional and psychological status (Salciccia et al., 2021). The onset of psychological distress is a chronic threat that peaks in severity and has a significant impact on the patient health status (Maggi et al., 2019). Psychological factors also may be the key factors of ED in CP/CPPS patients (Chen et al., 2015). In fact, CP/CPPS patients are often accompanied by psychological disorders due to pain, LUTS and psychosocial problems, which tend to decrease sexual activity and erectile function (Wang et al., 2016). Chronic pain in CP/CPPS patients and LUTS in aging men with benign prostatic hyperplasia (BPH) can definitely affect erectile function and these links are already suggested (Tran and Shoskes, 2013; De Nunzio et al., 2018). However, very few researches have disclosed the relationship of psychological stress with LUTS and ED in younger patients with CP/CPPS, as compared to BPH.

Sustained psychological stress exerts adverse effects on mental and physical health (Silverman and Deuster, 2014). It is known to cause physiological distress, leading to body balance perturbations associated with various metabolic and immune dysfunctions (Cui et al., 2021). The metabolic syndrome and its components predispose to obesity, diabetes and cardiovascular disorders (Licht et al., 2010), and generally, these metabolic disorders are strongly linked to ED (McMahon, 2019). Moreover, clinicians have observed that stress worsens the symptoms of prostatitis, and chronic stress in rats can even specifically induce histological inflammation of the prostate (Anderson et al., 2008).

Psychological stress triggers a cascade of pathophysiological events mediated by the autonomic nervous system (ANS) and the hypothalamic–pituitary–adrenal (HPA) axis (Anderson et al., 2008; Marques et al., 2010; Mazgelytė et al., 2021). These two neural stress systems coordinate the responses of many other physiological systems to stressors, including the immune and cardiovascular systems, allowing the body to return to homeostasis (Marques et al., 2010). Traditionally, HPA axis activity has been assessed by measuring its end-product cortisol, whereas ANS activity has been assessed by heart rate variability (HRV; Mazgelytė et al., 2021). Dysregulation of the HPA axis or ANS can significantly disrupt homeostasis, causing cacostasis or allostasis, with a range of clinical manifestations. This could be a potential mechanism contributing to the pathogenesis in both CP/CPPS and ED.

Thus, to expand our understanding of the complexities of psycho-neuro-endocrine interactions linking the mind and body in CP/CPPS patients with LUTS and ED, in this study, we test the hypothesis that LUTS-dominated CP/CPPS and ED are associated with psychological dysfunction and alterations in the HPA axis and ANS pathway.

## Materials and methods

### Participants

Between March 2016 and June 2021, CP/CPPS and ED patients who visited the Urology Male Clinic of our hospital were enrolled. Healthy men as controls were recruited from advertisements in the community.

Inclusion criteria were: CP/CPPS were diagnosed according to the National Institutes of Health Chronic Prostatitis Symptom Index (NIH-CPSI) items (Li et al., 2021), including frequent urination, urinary discomfort, perineal pain and discomfort, and varying degrees of sexual dysfunction for at least 3 months; ED diagnosed based on diagnostic criteria of the National Institutes of Health, with a course of disease longer than 3 months and a score of 5–21 in the International Index of Erectile Function-5 (IIEF-5) system (Li et al., 2021), no conscious penile erection, poor hardness or non-lasting erection, and inability to complete normal sexual life; >18 years of age; married or long-term regular sexual partners; genital examination showing no obvious developmental deformity; normal development of secondary sexual characteristics.

Exclusion criteria were: other lower urinary tract diseases such as urinary tract infections, tuberculosis, renal calculus, BPH, prostate cancer, urethral strictures, bladder tumors, and neurogenic bladder; sexual dysfunction or abnormal sex hormone test; psychosis; peripheral vascular disease; diabetes; spinal cord injury; coronary heart disease; hypertension; a history of alcohol or drug abuse. The patients did not use drugs affecting sexual function in the past 6 months, and were not administered drugs or other methods for ED treatment within 3 months.

### Study design and procedure

A cross-sectional study design was applied for the psychometric assessments of qualified subjects utilizing several self-rated questionnaires. We recruited 393 men with CP/CPPS, 229 ED patients, and 58 healthy controls. An attending urologist approached them and offered them a free evaluation and discussion of test results to participate in the study. With the help of the same urologist, all subjects were asked to complete a series of self-administered symptomatic and psychological questionnaires. According to the scores of the International Prostate Symptom Score (IPSS), NIH-CPSI and IIEF-5 (Ito et al., 2020), qualified subjects were divided into 4 groups. Subjects in the CP/CPPS+ED group were CP/CPPS cases with no pain symptoms, but accompanied by LUTS and ED. The CP/CPPS group encompassed CP/CPPS cases accompanied by only LUTS, with no pain symptoms or ED. The ED group comprised ED cases with no CP/CPPS symptoms. Healthy individuals were assessed as the normal control group. Grouping criteria are shown in Table 1.

**Table 1:** Grouping criteria.

Group	CP/CPPS+ED	CP/CPPS	ED	Control
NIH-CPSI	≥5	≥5	<5	<5
IPSS	≥8	≥8	0	0
IIEF-5	<12	≥22	<12	≥22

NIH-CPSI, National Institutes of Health Chronic Prostatitis Symptom Index; IPSS, International Prostate Symptom Score; IIEF-5, International Index of Erectile Function-5; CP/CPPS, chronic prostatitis/chronic pelvic pain syndrome; ED, erectile dysfunction.

Changes in HRV and cortisol secretion were further explored as markers of the ANS and HPA axis, we took the salivary cortisol level as the main observation index, the sample size was estimated, and each group needed at least 21 cases. Therefore, 25 age-matched cases were finally included per group. Saliva samples were collected successively from participants at 0, 15, 30, and 60 min after they woke up in their home, according to gold standards for cortisol awakening response (CAR; Stalder et al., 2016). On the days CAR samples were taken, saliva samples were also taken to monitor diurnal cortisol rhythms at 3, 6, 9, and 12 h after awakening. Our study included only participants who accurately completed saliva sample collection and provided sufficient amounts of saliva for cortisol measurements. Then, Subjects came to the clinic with collected saliva samples in 3 consecutive days after being asked to avoid strenuous exercise on the 4th day prior to the 24-h electrocardiogram (ECG) testing. The study protocol was approved by the Medical Ethics Committee of our hospital, and all participants provided informed consent.

### Questionnaires

All subjects used self-reported questionnaires, including demographic data, symptom assessment, and psychometric measures. Demographic data including age, employment, marital status, and education level were collected for all participants. In addition, patients reported on the duration of their CP/CPPS or ED symptoms.

CP/CPPS symptoms were assessed with the NIH-CPSI (Li et al., 2021), a 9-item index, consists of three domains including 2 items of urinary symptoms, 4 of pain, and 3 of QoL. The severity of LUTS was assessed by the IPSS (Ito et al., 2020), which contains 7 questions on urinary function (scored 1–5, with a higher score signifying worsening symptoms). ED was measured by the IIEF-5 (Li et al., 2021), which consisted of five questions, each scored on a scale of 0 to 5.

Instruments to measure psychological traits were applied to all participants and are described in the subsequent discussions. The Beck Anxiety Inventory (BAI) is a 21-item scale that assesses symptoms of anxiety which are minimally shared with depression (Bagheri et al., 2021). The score ranges from 0 to 63. The Perceived Stress Scale (PSS) is a 14-item questionnaire that examines the cognitive appraisal of stressful events during the past month (Khafagy et al., 2020). The PSS assesses unpredictability, burden overload and lack of control, and includes direct inquiries about current levels of experienced stress. The PSS score ranges from 0 to 56. The Type A Personality Test (TAPT) classifies personalities according to their response to stress (Vergès et al., 2021). A score between 85 and 154 indicates Type A behavior pattern. The Symptom Checklist 90 (SCL-90; Dang et al., 2021), a multidimensional, self-administered, likert-type instrument, is designed to assess a wide range of psychological problems. This questionnaire consists of 90 items divided into the following 10 factors: obsessive compulsiveness, somatization, interpersonal sensitivity, depression, hostility, anxiety, phobic anxiety, psychoticism, paranoid ideation, and others (sleep and diet). A Likert 5-point scale is used to rate the severity of each question, ranging from 0 (not at all) to 4 (extremely).

### Salivary cortisol

#### Collection of saliva

Participants were given labeled 1.5-ml sterile Eppendorf tubes and adjustable alarm clocks to collect saliva samples, and they were asked not to eat, drink, chew gum, smoke, brush their teeth or use mouthwash for 30 min before collection. Saliva samples were taken at eight points over three consecutive days, with strict reference to the time of morning awakening: 0, 15, 30, and 60 min after awakening, followed by four more samples at 3-h intervals throughout the day. To ensure compliance with saliva collection procedures, we adjust the alarm clock to beep at designated times for saliva collection. In addition, on the night before each CAR measurement, participants’ cell phone numbers were collected and a notification message was sent to them. This non-invasive technique was used for home or work collection to minimize disruption to normal daily life. At the end of saliva collection, all samples were placed in an insulated cold bag and taken back to the clinic. After centrifugation (3,000 rpm/5 min), the supernatants of saliva were kept at-70 °C for further analysis.

#### Analysis of cortisol

The concentration of free cortisol in saliva was assayed in duplicate using a cortisol radioimmunoassay (^125^I) kit (Beijing North Institute of Biotechnology, Beijing, China), according to the manufacturer’s specifications. Assay sensitivity was estimated at 0.1 nmol/l. The intra-and inter-analysis variance coefficients were less than 6 and 10%, respectively. Cortisol responses were assessed using two methods: CAR and diurnal profile. CAR was assessed using samples collected at 0, 15, 30, and 60 min after awakening, while the diurnal variation of cortisol in 8 time points was also studied. As a measure of CAR, the areas under the curve with respect to increase (AUCi) and ground (AUCg) were calculated using the formula of Pruessner et al. (2003). AUCg value reflected the total cortisol secretion within 1 h after awakening, while AUCi indicated changes (positive or negative) in cortisol concentration, thus signifying HPA axis reactivity and response to arousal stress. The diurnal spectrum was evaluated by the diurnal cortisol slope (DCS) and diurnal cortisol AUCg. DCS represented the change in cortisol secretion across the day, which was estimated by fitting a line that best matched all cortisol values (Hall et al., 2019). The diurnal AUCg, which was calculated using aforementioned trapezoidal method (Pruessner et al., 2003), showed the total salivary cortisol secretion and total HPA axis activity during the day.

### Heart rate variability

We used wireless Holter (BMS Century 3,000; Biomedical Systems, St. Louis, MO, United States) monitoring to obtain 24-h electrocardiogram (ECG) recordings suitable for HRV analysis. The participants followed their regular daily life and completed a time-activity diary during the recording time. To avoid the influence of circadian cycle, all measurements were scheduled to start at 7–8 a.m. and end at 24 h later. An ECG analysis software (CardioScan 12 Satellite; DM Software Inc., Beijing, China) was then used to analyze heart rate variability (HRV) monitored every 24 h. Arrhythmias and noise were automatically identified and filtered by the same software prior to analysis of HRV parameters and then validated or modified, if necessary, by a trained cardiologist. We selected four frequency-domain parameters, including very low frequency power (VLF, 0.01–0.04 Hz), low frequency power (LF, 0.04–0.15 Hz), high frequency power (HF, 0.15–0.4 Hz), and LF/HF ratio, as well as four time-domain parameters, including standard deviation of normal-to-normal R-R intervals (SDNN), root mean square of the successive differences of R-R intervals (rMSSD), the proportion of number of pairs of successive R-R intervals that differ by more than 50 ms divided by total number of R-R intervals (pNN50) and standard deviation of the average R-R intervals calculated over 5 min (SDANN).

### Statistical analyses

SPSS 17.0 (SPSS Inc., Chicago, IL, United States) was used for all statistical analyses. All continuous variables were tested for Kolmogorov–Smirnov normality to show their distribution. Continuous variables were expressed as median and range or mean ± standard deviation (SD), depending on the (non-) normal distribution of the measured variables. Discontinuous variables were described as number (percentage). One-way or two-way analysis of variance (ANOVA) combined with LSD-T test was used to compare normal distribution continuous variables, and Kruskal-Wallis test was used to compare non-normal variables. Chi-square test or Fisher’s exact test was used for categorical variables. Pearson correlation coefficient was used to analyze the correlation between symptoms and psychological scores. In addition, the IIEF-5 scores were adjusted for age because of the ED known to be age dependent. *p* < 0.05 was considered statistically significant. GraphPad Prism 8.0 software (La Jolla, CA, USA) was used to visualize the statistical results, and variables were normalized as appropriate. Normally distributed and continuous variables were standardized as the min-max normalization. Because of positively skewed distributions of salivary cortisol and HRV frequency-domain parameters, log-transformed variables were used.

## Results

### Demographic and symptomatic characteristics

Of 680 men who met inclusion criteria, 561 men completed the survey, with a response rate of 82.5%. 119 (17.5%) men discontinued the study because of consent withdrawal, incomplete information and other reasons. There were 95 (16.9%) patients in CP/CPPS+ED group, 290 (51.7%) patients in CP/CPPS group, 124 (22.1%) patients in ED group and 52 (9.3%) men in control group.

The ages of all subjects ranged from 18 to 49 (33.1 ± 7.6) years. The four groups of men were successfully matched for age, with a median age of 32 years (*p* = 0.186), and had similar body mass index (BMI; *p* = 0.29). CP/CPPS+ED patients had the longest symptom duration, whereas ED group had the shortest (*p* = 0.032). Distributions of occupational status (*p* < 0.0001), marital status (*p* < 0.0001) and education level (*p* < 0.0001) differed significantly across groups. The majority of the subjects in all 4 groups were employed, highly educated and were living together with their partners. Symptomatically, scores for NIH-CPSI, IPSS and IIEF-5 were consistent with the grouping criteria and significantly different in 4 groups (*p* < 0.0001 for all). Table 2 presents detailed characteristics of each group.

**Table 2:** Demographic, symptomatic, and psychological characteristics of participants.

Characteristic	CP/CPPS+ED	CP/CPPS	ED	Control	*p* Value*
Sample size, n	95	290	124	52	N/A
Age (years), mean ± SD	33.4 ± 7.8	33.5 ± 7.4	31.8 ± 7.7	32.7 ± 7.6	0.186
BMI (kg/m^2^), mean ± SD	24.0 ± 3.1	23.5 ± 2.8	23.7 ± 2.0	24.0 ± 2.4	0.290
Symptom duration (months), mean ± SD	33.4 ± 23.2	32.8 ± 17.9	27.8 ± 17.6	N/A	0.032
Employment, n(%)					<0.001
Student	3(3.2)	63(21.7)	4(3.2)	19(36.5)	
Employed	62(65.3)	136(46.9)	54(43.6)	15(28.9)	
Unemployed or retired	30(31.5)	91(31.4)	66(53.2)	18(34.6)	
Marital status, n(%)					<0.001
Never married/single	0(0)	17(5.9)	0(0)	5(9.6)	
Married/cohabiting	86(90.5)	239(82.4)	122(98.4)	43(82.7)	
Divorced or separated	9(9.5)	34(11.7)	2(1.6)	4(7.7)	
Educational level, n(%)					<0.001
Less than high school	44(46.3)	83(28.6)	15(12.1)	10(19.2)	
High school graduate/vocational school	20(21.1)	80(27.6)	34(27.4)	19(36.5)	
College graduate or higher education	31(32.6)	127(43.8)	75(60.5)	23(44.3)	
Symptom scores, mean ± SD					
NIH-CPSI	13.0 ± 4.2	13.3 ± 4.0	0.7 ± 0.8	1.1 ± 1.0	<0.001
Pain score	0	0	0	0	N/A
Urinary symptom score	4.4 ± 2.6	5.3 ± 2.5	0.5 ± 0.7	0.6 ± 0.7	<0.001
QoL impact score	8.6 ± 2.4	8.0 ± 2.4	0.2 ± 0.5	0.5 ± 0.7	<0.001
IPSS	14.8 ± 5.4	15.4 ± 6.1	0	0	<0.001
IIEF-5	8.9 ± 2.0	22.9 ± 0.9	8.3 ± 2.3	23.1 ± 0.9	<0.001
Psychological scores, mean ± SD					
BAI	13.4 ± 14.5	12.0 ± 9.9	9.5 ± 10.5	4.8 ± 5.0	<0.001
PSS	18.8 ± 5.0	15.9 ± 4.8	16.9 ± 5.8	14.0 ± 5.9	<0.001
TAPT	48.0 ± 8.3	45.1 ± 9.1	47.2 ± 8.5	46.7 ± 8.8	0.016
SCL-90	153.5 ± 60.3	131.5 ± 35.0	134.7 ± 47.9	114.8 ± 24.1	<0.001

*SD*, standard deviation; BMI, body mass index; NIH-CPSI, National Institutes of Health Chronic Prostatitis Symptom Index; QoL, quality of life; IPSS, International Prostate Symptom Score; IIEF-5, International Index of Erectile Function-5; BAI, Beck Anxiety Inventory; PSS, Perceived Stress Scale; TAPT, Type A Personality Test; SCL-90, Symptom Checklist 90; CP/CPPS, chronic prostatitis/chronic pelvic pain syndrome; ED, erectile dysfunction.

* *p* value from ANOVA for age, BMI, symptom duration, symptom and psychological scores, and from the Chi-square test or Fisher’s exact test for all other variables. N/A = not applicable.

### Psychological scores

**Figure 1:**
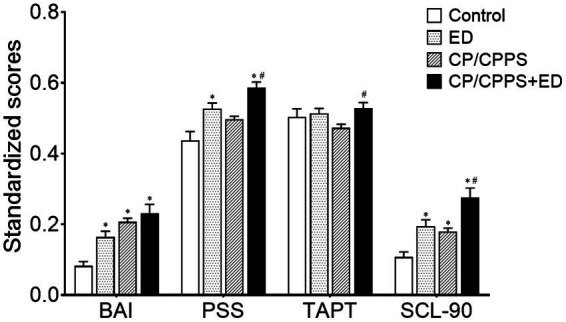
Standardized psychological scores in 4 groups. All data were standardized as the min-max normalization. *p < 0.01 versus the control group, ^#^*p* < 0.01 versus CP/CPPS group.

The 4 groups were distinguishing on psychological variables. The BAI and SCL-90 scores were significantly higher in the CP/CPPS+ED, CP/CPPS and ED groups compared with the control group (*p* < 0.01). The PSS scores of both groups with ED were significantly higher than the control group (*p* < 0.01). There was no difference in all psychological parameters between CP/CPPS+ED and ED groups (*p* > 0.01), CP/CPPS and ED groups (p > 0.01). Compared with the CP/CPPS group, the differences of PSS, SCL-90 and Type A behavior pattern scores were statistically significant in patients with both CP/CPPS and ED (*p* < 0.01). Psychometric evaluation scores are shown in Table 2 and Figure 1.

### Correlation of IPSS with NIH-CPSI, BAI, PSS, and SCL-90 in the CP/CPPS patients

**Figure 2:**
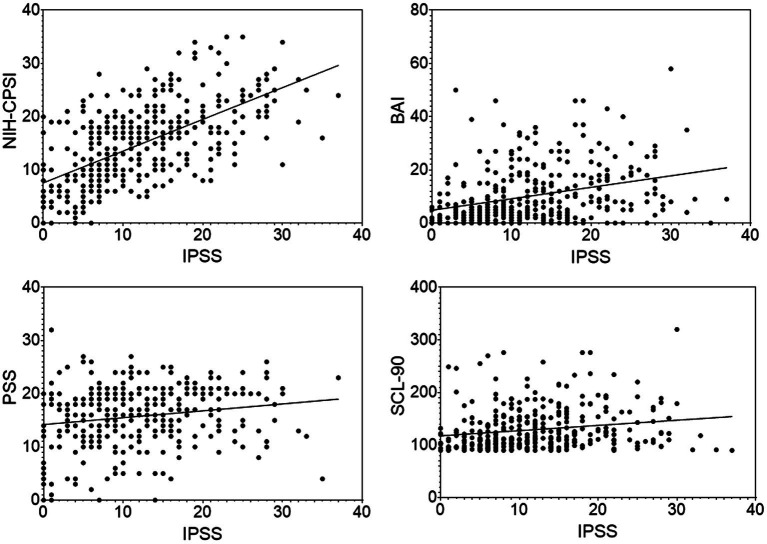
Scatter plots of the IPSS scores with the NIH-CPSI, BAI, PSS, SCL-90 scores in the CP/CPPS patients. Data points represent individual participants.

The IPSS scores were shown to have significantly positive correlations with BAI (*r* = 0.32, *p* < 0.0001), PSS (*r* = 0.18, *p* < 0.01) and SCL-90 (*r* = 0.19, *p* < 0.01) in the CP/CPPS patients (Figure 2). The urinary symptom, QoL, and sum domains of the NIH-CPSI were also shown to have a significantly positive correlation with the IPSS score in the CP/CPPS patients (including CP/CPPS and CP/CPPS+ED groups. *r* = 0.67, *p* < 0.0001; *r* = 0.49, *p* < 0.0001; and *r* = 0.61, *p* < 0.0001, respectively).

### Correlation of IIEF-5 with QoL, BAI, PSS, and SCL-90 in all subjects

**Figure 3:**
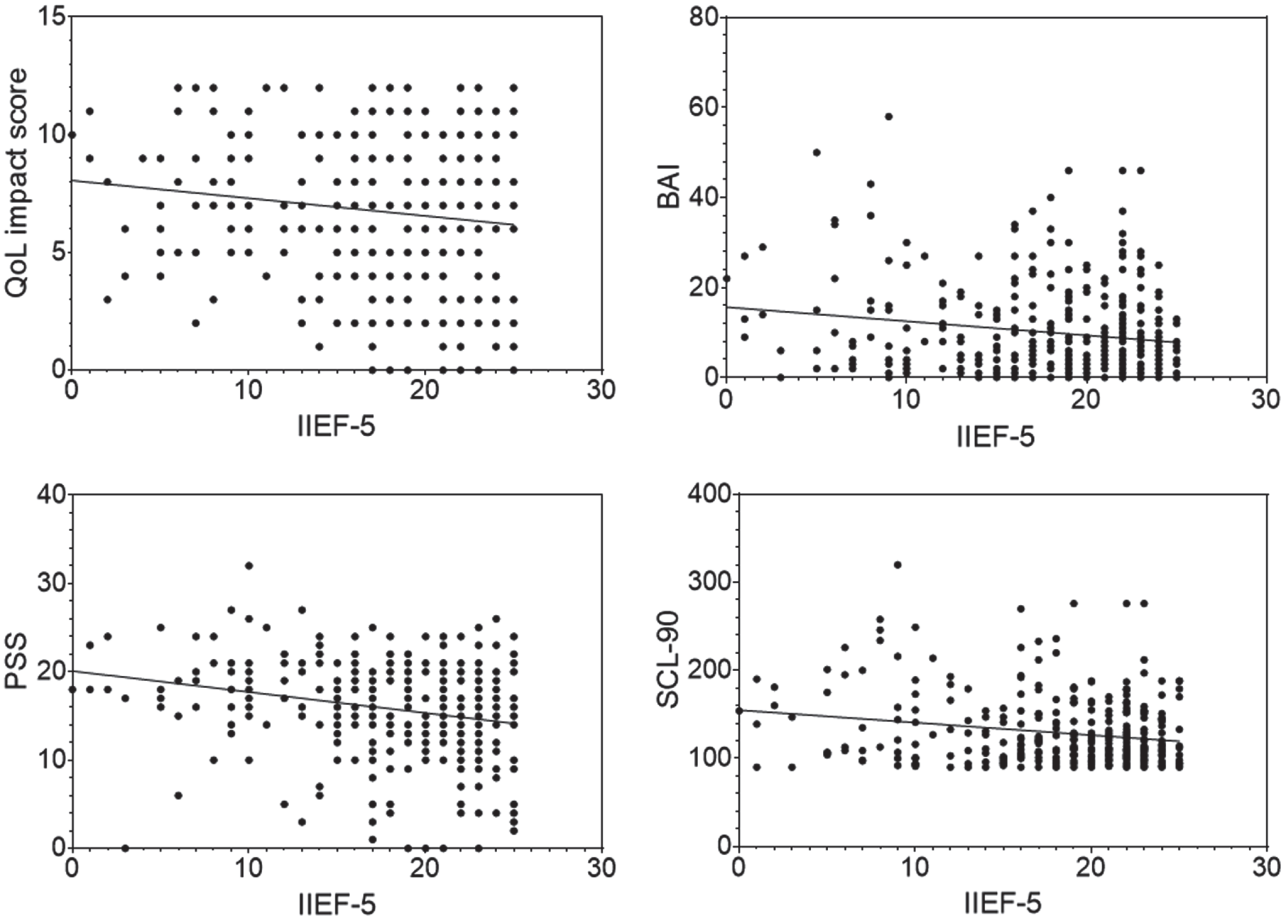
Scatter plots of the correlations of IIEF-5 scores against QoL, BAI, PSS, SCL-90 scores in all subjects. Data points represent individual participants.

For all participants, the IIEF-5 scores were shown to have significantly negative correlations with BAI (*r* = −0.17，*p* < 0.001), PSS (*r* = −0.25，*p* < 0.0001), SCL-90 (*r* = −0.20，*p* < 0.001) and QoL score in NIH-CPSI (*r* = −0.14，*p* = 0.0075; Figure 3). No significant correlations were found between the urinary symptom score or the total score of the NIH-CPSI and IIEF-5 score (*r* = 0.07, *p* = 0.1943; *r* = −0.08, *p* = 0.1337).

### Salivary cortisol

**Figure 4:**
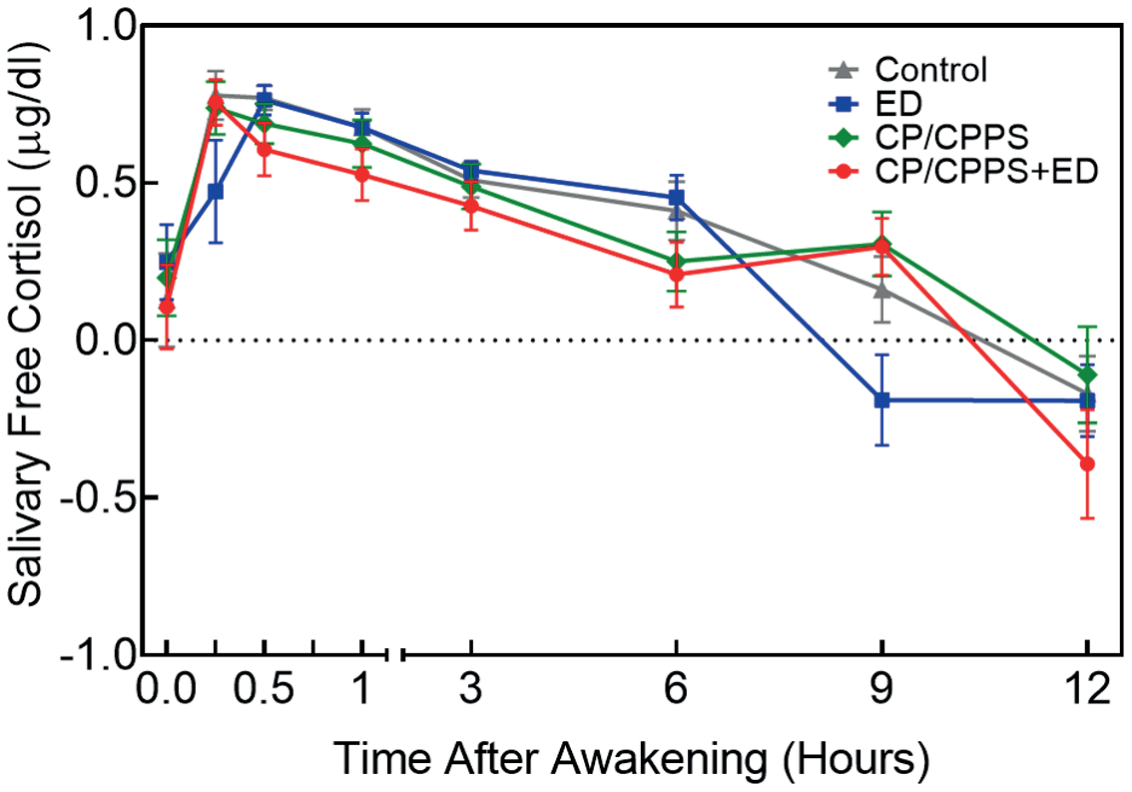
Mean diurnal salivary cortisol concentrations±standard error of means (SEM) at 8 time points in 4 groups. All values were log-transformed.

Table 3 and Figure 4 showed the differences in diurnal salivary cortisol levels in 4 groups. Salivary cortisol data were log-transformed prior to statistical analysis, but are presented as true values in Table 3 without log-transformed for clarity. The results of two-way ANOVA showed that cortisol levels across time had a significant main effect (confirming that all participants displayed a typical CAR; *F*_7,768_ = 40.98, *p* < 0.0001), but did not show a group effect (showing that the mean values of salivary cortisol concentrations did not differ between groups at all time points; *F*_3,768_ = 1.467, *p* = 0.2222). No significant interaction was found between time and group variables (*F*_21,768_ = 1.489, *p* = 0.0731).

**Table 3:** Salivary cortisol and HRV parameters in 4 groups.

Parameters	CP/CPPS+ED	CP/CPPS	ED	Control	*p* Value*
Sample size, *n*	25	25	25	25	N/A
Age (years), mean ± SD	34.8 ± 5.1	34.2 ± 6.7	34.2 ± 4.2	33.6 ± 7.6	0.920
CAR (μg/dl), median (range)					
0 min (awakening)	1.2 (0.11–13.08)	2.8 (0.11–13.08)	4.5 (0.13–6.84)	3.1 (0.01–13.08)	0.937
+15 min	6.2 (0.10–13.40)	5.0 (0.28–46.59)	7.0 (0.09–12.47)	5.7 (0.28–33.04)	0.873
+30 min	4.7 (0.32–12.80)	5.0 (0.31–12.37)	6.3 (2.15–12.05)	5.4 (2.15–12.05)	0.040
+60 min	4.2 (0.90–37.42)	4.3 (0.50–51.39)	4.6 (0.77–13.04)	4.8 (0.77–35.98)	0.002
Diurnal cortisol (μg/dl), median (range)					
+3 h	3.2 (0.28–11.38)	3.2 (0.19–11.38)	3.3 (1.11–11.38)	3.6 (0.19–11.38)	0.653
+6 h	2.2 (0.03–6.44)	2.2 (0.03–26.71)	3.1 (0.14–7.50)	2.5 (0.03–26.71)	0.003
+9 h	1.7 (0.13–27.45)	2.0 (0.13–40.21)	1.3 (0.02–16.59)	1.5 (0.13–27.45)	0.000
+12 h	0.5 (0.01–19.34)	1.9 (0.01–5.92)	0.8 (0.01–4.87)	1.0 (0.01–5.10)	0.044
CAR AUCi (μg·min/dl), median (range)	113.7 (−550.52–772.40)	103.8 (−550.52–1377.43)	114.5 (−124.18–461.48)	124.0 (−550.52–1107.44)	0.999
CAR AUCg (μg·min/dl), median (range)	306.2 (25.33–866.41)	320.9 (92.98–1544.83)	365.5 (92.98–577.13)	350.8 (92.98–1108.28)	0.457
Diurnal AUCg (μg·min/dl), median (range)	2111.0 (666.45–9218.93)	2067.3 (729.43–12963.95)	2252.3 (805.75–4833.07)	2141.6 (554.29–10346.03)	0.963
DCS (μg/dl/h), median (range)	−0.02 (−1.04–0.96)	−0.04 (−1.04–0.96)	−0.21 (−0.48--0.11)	−0.05 (−1.04–0.18)	0.077
Frequency-domain HRV					
VLF (ms^2^), median (range)	3402.0 (505.0–6623.0)	1936.5 (285.0–10053.0)	2837.5 (146.0–6623.0)	2376.0 (240.0–5428.0)	0.415
LF (ms^2^), median (range)	908.5 (293.0–5149.0)	1017.0 (153.0–5924.0)	680.5 (121.0–6289.0)	1893.0 (150.0–4876.0)	0.011
HF (ms^2^), median (range)	934.0 (112.0–2125.0)	1108.0 (82.1–9624.0)	622.0 (127.0–9624.0)	3845.0 (1179.0–7496.0)	<0.001
LF/HF, median (range)	1.6 (0.14–27.68)	0.8 (0.08–5.21)	0.7 (0.07–14.05)	0.6 (0.03–3.47)	0.039
Time-domain HRV, mean ± SD					
SDNN (ms)	137.2 ± 40.5	143.6 ± 64.4	133.3 ± 63.0	138.8 ± 55.9	0.935
SDANN (ms)	122.4 ± 45.6	123.7 ± 67.4	117.4 ± 68.4	121.0 ± 58.0	0.985
rMSSD (ms)	44.8 ± 15.1	51.0 ± 26.8	46.2 ± 24.8	55.9 ± 29.0	0.370
pNN50 (ms)	16.4 ± 9.3	17.1 ± 7.7	15.0 ± 7.6	21.4 ± 9.4	0.056

SD, standard deviation; CAR, cortisol awakening response; AUCi, area under the curve with respect to increase; AUCg, area under the curve with respect to ground; DCS, diurnal cortisol slope, HRV, heart rate variability; VLF, very low frequency; LF, low frequency; HF, high frequency; SDNN, standard deviation of all R-R intervals; SDANN, standard deviation of the average R-R intervals calculated over 5 min; rMSSD, root mean square of successive differences; pNN50, percent of R-R intervals differing more than 50 ms from each other; CP/CPPS, chronic prostatitis/chronic pelvic pain syndrome; ED, erectile dysfunction.

* *p* value from Kruskal–Wallis test for salivary cortisol, frequency-domain HRV values, CAR AUCi and AUCg, diurnal AUCg and DCS, and from ANOVA for time-domain HRV values. N/A = not applicable.

Post-hoc tests indicated that ED and control groups showed higher salivary cortisol concentrations than CP/CPPS+ED group at 30 min (*p* = 0.006 and *p* = 0.003), 1 h (p = 0.007 and *p* = 0.011) and 6 h (*p* = 0.001 and *p* = 0.013) after awakening. ED and control groups also showed higher salivary cortisol concentrations than CP/CPPS group at 6 h after awakening (*p* = 0.004 and *p* = 0.040). The results at 9 h after awakening indicated that CP/CPPS+ED, CP/CPPS, control groups had significantly greater levels of cortisol than ED group (*p* < 0.001, *p* < 0.001 and p = 0.001, respectively). At the last time point, results revealed that CP/CPPS patients had greater cortisol levels than CP/CPPS+ED patients (*p* = 0.037). No differences in cortisol levels were found at other time points between groups.

**Figure 5:**
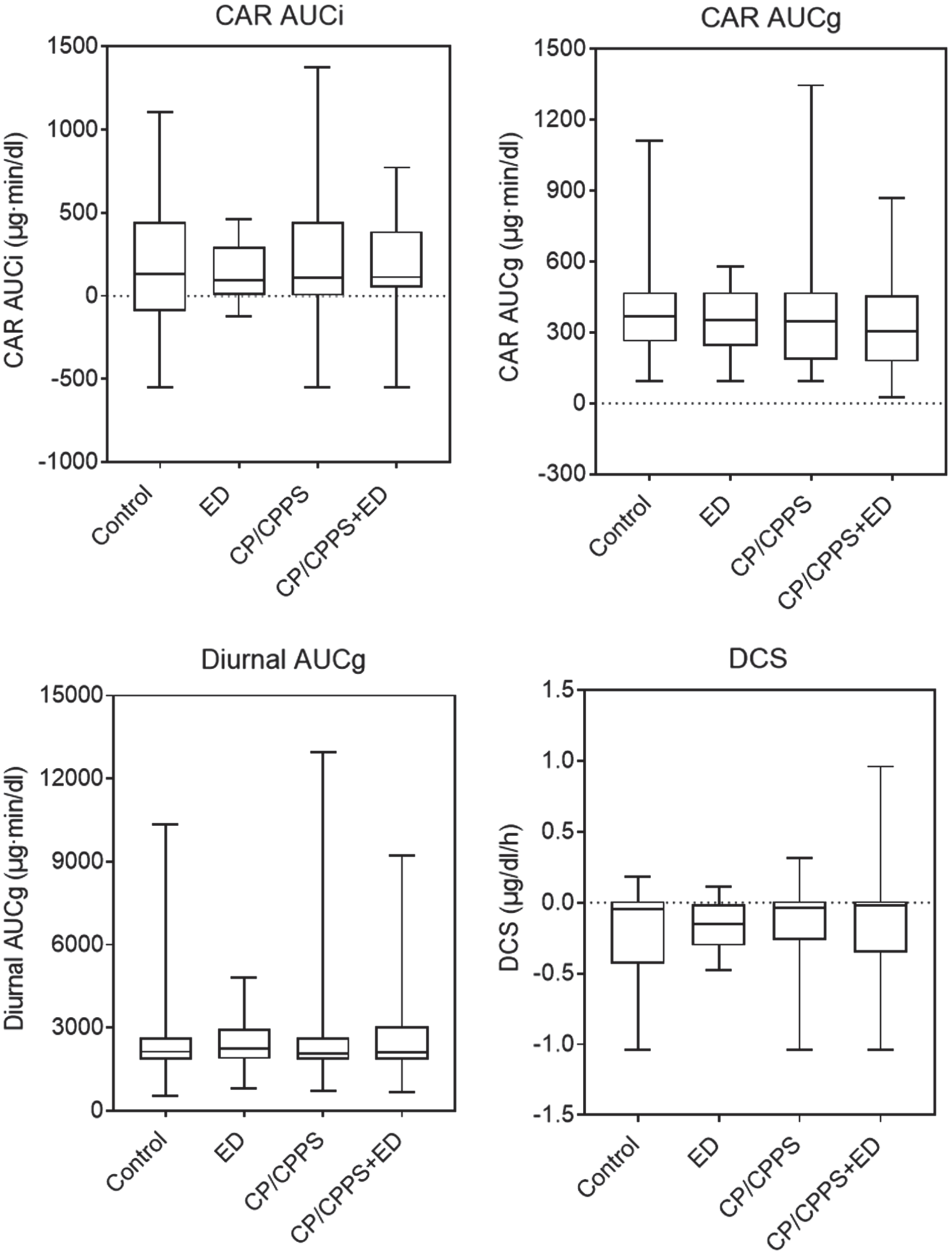
Waking and diurnal salivary cortisol parameters in 4 groups. Line in box indicates median. Boundary closest to *X*-axis indicates 25th percentile and farthest from *X*-axis indicates 75th percentile. Whiskers indicate minimum and maximum.

As shown in Table 3 and Figure 5, the dynamic changes in cortisol in the morning as showed by CAR AUCi and the total secretion of salivary cortisol over the first hour after awakening as indicated by CAR AUCg did not differ significantly across groups (*p* = 0.999 and *p* = 0.457, respectively). Similarly, the Kruskal-Wallis test showed no significant differences between groups in diurnal cortisol rhythm, including diurnal AUCg (*p* = 0.963) and DCS (*p* = 0.077).

### HRV analysis

**Figure 6:**
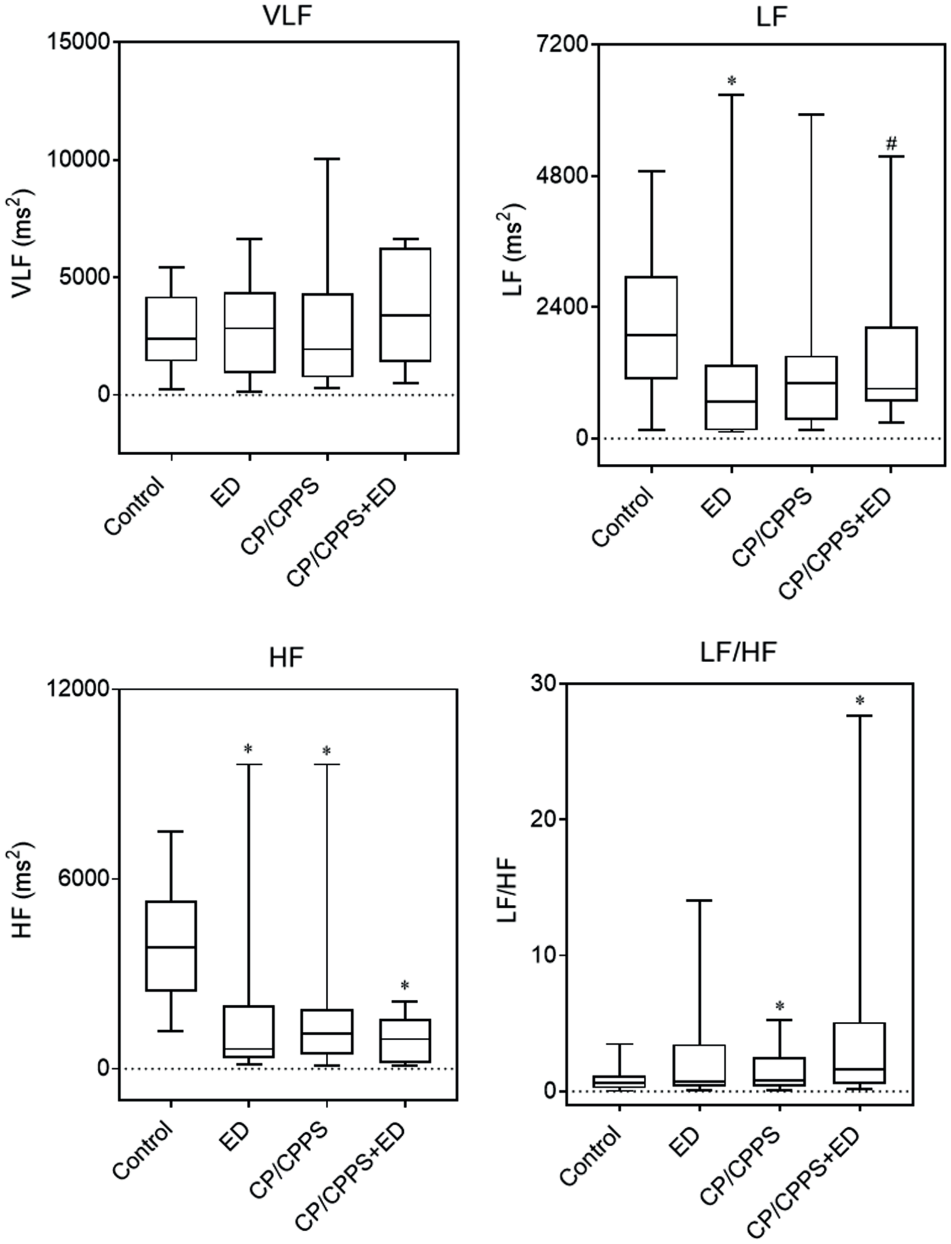
HRV frequency-domain parameters in 4 groups. Line in box indicates median. Boundary closest to *X*-axis indicates 25th percentile and farthest from *X*-axis indicates 75th percentile. Whiskers indicate minimum and maximum. *p < 0.01 versus the control group. ^#^*p* < 0.05 versus the ED group.

**Figure 7:**
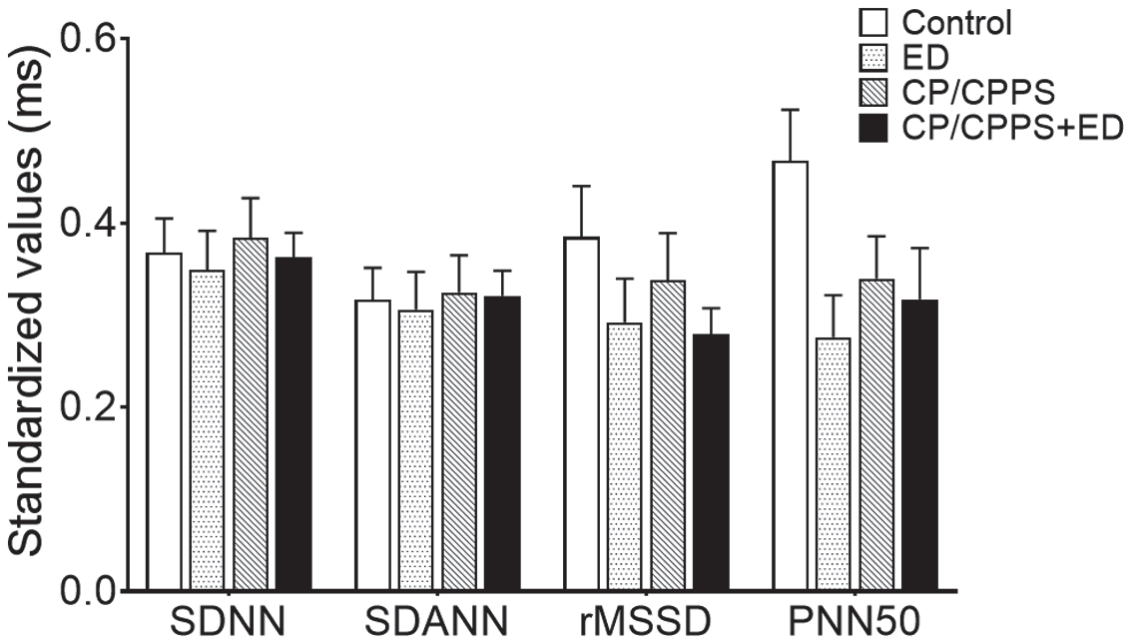
Standardized HRV time-domain parameters in 4 groups. All time-domain values were standardized as the min-max normalization.

In the frequency domain analysis, the LF of ED group was significantly lower than that of CP/CPPS+ED group (*p* = 0.044) and control group (*p* = 0.005), HF power was significantly higher in healthy controls compared to patients with ED (*p* < 0.001), CP/CPPS (*p* < 0.001) and CP/CPPS+ED (*p* < 0.001), and the CP/CPPS+ED patients had significantly higher LF/HF ratio than the controls (p = 0.001; Table 3 and Figure 6). No statistically significant differences in changes among groups were seen in any time-domain variables of HRV during the study (all *p* > 0.05; Table 3 and Figure 7).

## Discussion

The range of ED prevalence varied widely, probably reflecting differences in the age of the study population. The prevalence of ED under the age of 49 in the general population was 1–10% (Zhang et al., 2016). In 370 Chinese men with chronic prostatitis, the prevalence of ED as assessed by IIEF-5 score was 35.1% (Hao et al., 2011). Furthermore, the incidence of ED in men with BPH-associated LUTS ranged from about 35 to 95% and increased with age and LUTS severity (Calogero et al., 2019). In China, ED were found in 82.16% of LUTS patients aged over 40 (Liang et al., 2014). Therefore, High prevalence of ED was found in men with CP/CPPS or LUTS/BPH compared to the general population. Our data determined that the prevalence of ED in patients with CP/CPPS dominated by LUTS was 24.7% (95 out of 385 patients had ED), which is similar to previous findings (Chung et al., 2012; Tran and Shoskes, 2013; Chen et al., 2015; Fan et al., 2021), indicating that LUTS in CP/CPPS has a negative effect on erectile function.

The link between concomitant ED and LUTS in CP/CPPS patients might be psychological factors. Both CP/CPPS and ED patients suffered from considerable stress, depression, and anxiety (Chen et al., 2015). In this study, a combination of various questionnaires from the CP/CPPS+ED, CP/CPPS, ED, and Control groups, respectively, was used to systematically analyze each subject’s psychological symptoms and mental state. In addition to the PSS score of CP/CPPS group, the psychological symptoms such as depression, anxiety, somatization disorder, self-reported perceived stress and obsessive–compulsive behavior in the BAI, PSS, and SCL-90 domains of men in symptom groups were significantly higher than those of age-matched asymptomatic men. This result was not only consistent with our study expectation, but also falling in line with previous investigations with respect to psychometric assessments of CP/CPPS and ED (Anderson et al., 2008, 2009; Koh et al., 2014; Huri et al., 2016; Molina-Leyva et al., 2016). And it was found that CP/CPPS men with ED had statistically higher PSS, SCL-90 and TAPT scores than those without ED. Our results provided strong support for the hypothesis that ED is related to the psychological disorder of CP/CPPS patients. Multivariate logistic regression even demonstrated that psychological factors were independent risk factors for ED in CP/CPPS (Zhang et al., 2016). It was noteworthy that, however, in the current study, the personality of type A or B behavior pattern had few effects on LUTS and ED.

With regard to the correlation between psychological scores and severity of CP/CPPS and ED symptoms, the results of the present research showed that psychological scores, excepting TAPT due to incommensurability, were positively associated with IPSS and negatively associated with IIEF-5, suggesting that the severity of psychological stress was positively correlated with the severity of LUTS and ED. The IPSS and IIEF-5 each also had a significant correlation with QoL subscore of NIH-CPSI. In other words, the severity of both LUTS and ED was strongly and independently correlated to lower QoL (Zhao and Kim, 2019). It implied that the psychological impact from CP/CPPS might impair the overall QoL and that a low QoL could cause ED (Wang et al., 2016; De Nunzio et al., 2018).

Additionally, because psychosocial stress is often associated with chronic pain, a diathesis-stress model is emerging as the dominant theoretical perspective for describing the feed-forward relationship between chronic pain and psychopathology (Dersh et al., 2002). Recent studies have illustrated how the stress of coping with chronic pain activates and exacerbates semi-dormant but preexisting traits (diatheses), ultimately leading to diagnosable psychopathology (Dersh et al., 2002; Anderson et al., 2008; Msw and Rsw, 2017). These concepts might be equally applicable to interpreting the association between CP/CPPS with concurrent ED and psychological disturbances related to stress, depression, anxiety, and somatisation disorders.

According to the above speculation, in our next exploratory study, the possible dysregulation of HPA axis and ANS in 4 cohorts was examined to better understand the psychopathological mechanisms underlying the influence of psychological stress on CP/CPPS and ED. In CP/CPPS men with confirmed pelvic pain, previous study has provided evidence of HPA axis dysregulation reflected in augmented CAR (Anderson et al., 2008). Men with pelvic pain also had subtle altered HPA axis function in response to acute stress, namely, a delayed release of adrenocorticotropin concomitant with a relative adrenal hypersensitivity or adequacy, which might be the result of neuropsychological adjustments to chronic pain and stress on a central level (Anderson et al., 2009). With regard to the association between ED and the HPA axis, only a few studies have indirectly shown that drugs used to treat ED might affect the HPA axis. For instance, ginseng was involved in regulating the HPA axis and controlling hormone release, thus alleviating erectile dysfunction (Lee and Rhee, 2017). Moreover, erection-inducing apormorphine levels interfered with the HPA axis inhibitory feedback mechanism during interleukin-1β administration (Buller et al., 2003). Hence, in the current study, we removed the effect of pain on the HPA axis and mainly focused on the HPA axis activity in LUTS-dominated CP/CPPS patients with or without ED.

Measurement of salivary cortisol is considered a reliable and noninvasive indicator of HPA axis activity (Licht et al., 2010). The CAR and diurnal cortisol profile serve as useful indexes of adrenocortical activity, providing important information on HPA axis activation. Our results exhibited the following three typical characteristics. First, consistent with other research of HPA axis (Anderson et al., 2008; Hall et al., 2019), salivary cortisol had a circadian rhythm, with levels higher in the morning and falling throughout the day. Second, with the exception of slight fluctuations at several time points (statistically significant differences between some groups at 30 min, 1 h, 6 h, 9 h, 12 h after awakening), cortisol concentrations remained stable in the four groups. Third, measures of salivary cortisol (CAR AUCi, CAR AUCg, diurnal AUCg and DCS) did not significantly vary between the four groups. We noted that in contrast to pain symptom in CP/CPPS (Anderson et al., 2008, 2009), the chronically mental stress elicited by LUTS and ED did not necessarily lead to HPA axis dysregulation. Previous study has also reported no dysregulation of the HPA axis in patients with metabolic syndrome (Licht et al., 2010). However, most studies have described increased activation of the HPA axis during drastic physical changes (stressors), including chronic pain (Anderson et al., 2008, 2009), late-night eating (Uçar et al., 2021), acute ozone exposure (Wang et al., 2022), and anorexia nervosa (Paszynska et al., 2016). In the current study, the lack of change in HPA axis activity suggested that the HPA axis was desensitized and did not respond with hyperactivity. It seemed easier to be activated by a physical rather than mental stressor. Alternatively, the allostatic load induced by LUTS, ED, or both was probably not enough to trigger the threshold of HPA axis activation. It was also possible that the sample size was relatively small and did not change.

The activity of the ANS and the HPA axis coordinated with each other in response to stress (Anderson et al., 2008; Marques et al., 2010; Mazgelytė et al., 2021). As a marker of sympathovagal balance, HRV refers to a sensitive indicator of sympathetic and vagal activity (Kayacan et al., 2021; Mazgelytė et al., 2021). There are two main approaches for HRV analysis: time-domain analysis, which is a statistical calculation of how much variability exists; and frequency-domain analysis, the analysis of latent frequencies, gives information about autonomic balance and rhythm (Cho et al., 2011). Both methods have a high correlation with sympathetic and parasympathetic structures, which are known to accurately measure cardiac baroreflex activity and autonomic activity (Kayacan et al., 2021). Previous studies have shown that an imbalance in ANS activity is associated with many urologic diseases, including CP/CPPS (Cho et al., 2011), LUTS (Choi et al., 2010; Shim et al., 2018), and ED (Chen et al., 2009; Lee et al., 2011; Costa et al., 2021). Our findings found that none of the time-domain parameters differed significantly, but on frequency-domain analysis, all patients, including patients with CP/CPPS, ED, or both, exhibited significantly lower HF than healthy controls. Similar to our findings, The HF value of HRV in aging LUTS patients decreased, indicating that these patients may have an ANS imbalance, especially reduced parasympathetic activity (Choi et al., 2010). Cho et al. (2011) demonstrated that significantly lower HRV values (SDNN, rMSSD, VLF and HF) in CP/CPPS patients compared to healthy male volunteers and concluded that abnormal autonomic nerve function might be related to pelvic pain in CP/CPPS patients. The lower HRV was also a psychophysiological correlate of ED in middle-aged and elderly men (Kessler et al., 2019). Specifically, the HF values of ED patients were lower and LF/HF ratios were higher than those of the controls (Lee et al., 2011; Shim et al., 2018; Costa et al., 2021). In this study, we still observed that there were high LF/HF ratios in all patients, but only patients with both CP/CPPS and ED exhibited significantly higher LF/HF ratio, the increased LF/HF ratio indicating that sympathetic activity is higher than parasympathetic activity (Shim et al., 2018; Kayacan et al., 2021). As previously suggested, we reconfirmed the association of CP/CPPS and ED with sympathovagal imbalance, but only patients with both CP/CPPS and ED exhibited obvious influences on autonomic neural modulation by withdrawing parasympathetic activity (reduced HF index) and shifting sympathovagal balance toward a relative sympathetic prevalence (increased LF/HF ratio).

In summary, CP/CPPS and ED patients scored very high on most psychosocial variables. Except for the PSS score of CP/CPPS group, the psychological symptoms of depression, anxiety, somatization disorder, self-reported perceived stress and obsessive–compulsive behavior in BAI, PSS and SCL-90 domains of men in all symptom groups were significantly higher than those of asymptomatic men. Our results also showed that the PSS, SCL-90 and TAPT scores of CP/CPPS patients with ED were significantly higher than those of patients without ED. In the correlation between the psychological score and the severity of CP/CPPS and ED symptoms, the severity of psychological stress was positively correlated with the severity of LUTS and ED. However, in the HPA axis activity and HRV measurements, we found statistically significant changes only in the frequency domain analysis. All patients had significantly lower HF than healthy controls. We also observed higher LF/HF ratios in all patients, but only those with both CP/CPPS and ED showed significant increases. Data from stress system analysis showed that sympathetic vagal imbalance in ANS was associated with ED and LUTS in CP/CPPS, but HPA axis activity was not.

Because of the bidirectional dose–response relationship between LUTS/BPH and ED prevalence, as well as their common risk factors, both LUTS and erections improved after sildenafil treatment in men with ED and LUTS (De Nunzio et al., 2018; Kessler et al., 2019), from the aspect of etiology, it is likely that pathophysiology of LUTS may be similar to that of ED, and psychological factors are a strong contributor common to ED and LUTS (Calogero et al., 2019; Kessler et al., 2019). Interestingly, sympathetic nerve activity is the main mechanism of psychogenic ED (Chen et al., 2009), an increase in sympathetic tone is also responsible for LUTS (Choi et al., 2010; De Nunzio et al., 2018). Consequently, combined with our research, we can deduce that psychological factors ultimately contribute to both ED and LUTS in CP/CPPS, and the potential psychopathological mechanisms may be as follows. First, long-term mental stress, such as anxiety and depression, triggers a cascade of neuroendocrine changes that involve alterations in neurobiological stress systems, such as the ANS and HPA axis, especially increasing the activity of sympathetic nerves innervating the prostate sphincter, the bladder sphincter and detrusor, as well as the smooth muscle of the cavernous body of the penis (Chen et al., 2009; Choi et al., 2010; Cho et al., 2011; Lee et al., 2011; Shim et al., 2018; Costa et al., 2021). Then, the aforementioned smooth muscles are stimulated, causing “spasms” or “contractions” in the bladder neck, prostate urethra and penile cavernous body, finally resulting in obstructive urination symptoms and impotence, and in severe cases, urine reflux into the prostatic duct induces CP/CPPS.

The present study has some limitations. Firstly, this study was conducted in a single center; thus, our findings may not be generalizable. Secondly, the selection bias caused by subjective factors in data statistics and processing cannot be ruled out. Some people may be reluctant to see a doctor for CP/CPPS or sexual problems, and the patients in this study may be biased toward those with more severe and troublesome symptoms (Chung et al., 2012; Zhang et al., 2016). Thirdly, the study was also limited in measures of symptoms and psychological functioning due to its reliance on self-report questionnaires, no objective measures were included. Fourth, other uncontrollable factors might have confounded our results. For example, when comparing the ANS and HPA axis parameters among 4 groups, changes in both stress systems may be too small to be detected with our method and sample size. The timing of saliva samples was also a confounding factor due to the wide differences of real awakening times in participants (Hall et al., 2019). In addition, HRV is affected by a variety of physiological and environmental factors (Kayacan et al., 2021). Although drugs affecting autonomic activity were excluded, we could not control for individual mood, mental state, and environmental factors when measuring HRV (Lee et al., 2011). Finally, the intercorrelation of both stress systems was not investigated. Because of the observational study design, we were unable to determine the causal direction of the relationship between the stress system and patients’ psychosomatic status. It is necessary to further investigate the relationship between HPA axis, ANS, ED and CP/CPPS (Licht et al., 2010; Mazgelytė et al., 2021). Although our study has limitations, it is the first to evaluate the symptomatic and psychological scores, the HPA axis and the ANS together in LUTS-dominated CP/CPPS patients with and without ED and ED patients.

## Conclusion

Except for confirming the strong associations between ED and LUTS in CP/CPPS men, our data find that most psychological stress, mainly quantified by the BAI, PSS and SCL-90 scores, show significantly higher levels in CP/CPPS and ED patients when compared to healthy controls. The severity of LUTS and ED positively correlates with the severity of psychological stress and the lower QoL subscore of NIH-CPSI. Furthermore, from an overall point of view, the ANS dysregulation is associated with ED and LUTS in CP/CPPS, whereas HPA axis activity is not. Especially for patients with both CP/CPPS and ED, a decrease in parasympathetic and increase in sympathetic activity are observed and could therefore partly be involved in the pathogenesis of LUTS and ED. On the basis of our results, this study contributes to a better understanding of the complexity of psycho-neuro-endocrine interactions linking the mind and body in CPPS patients with LUTS and ED. Large-scale, longitudinal and multi-center investigations are merited to confirm and extend our findings.

## Data availability statement

The original contributions presented in the study are included in the article/supplementary material, further inquiries can be directed to the corresponding author.

## Ethics statement

The studies involving human participants were reviewed and approved by Institutional Review Board of Tongji Hospital, Tongji Medical College, Huazhong University of Science and Technology. The patients/participants provided their written informed consent to participate in this study.

## Author contributions

SW, JL, LJ, and JB contributed to conception and design of the study. ML, BH, SY, YC, and XD collected clinical data and specimens. ML, JY, and XL analyzed the data. JB wrote the first draft of the manuscript. TW, YC, and LG wrote sections of the manuscript. All authors contributed to the article and approved the submitted version.

## Funding

This work received support from the Hubei Province Health and Family Planning Scientific Research Project (WJ2021F102) and the National Natural Science Foundation of China (81974092).

## Conflict of interest

The authors declare that the research was conducted in the absence of any commercial or financial relationships that could be construed as a potential conflict of interest.

## Publisher’s note

All claims expressed in this article are solely those of the authors and do not necessarily represent those of their affiliated organizations, or those of the publisher, the editors and the reviewers. Any product that may be evaluated in this article, or claim that may be made by its manufacturer, is not guaranteed or endorsed by the publisher.
